# Contemporary Theory and Research

**Published:** 1893-02-18

**Authors:** 


### CONTEMPORARY THEORY AND RESEARCH.

### ELECTRICITY FOR CHOLERA.

We extract the following from a report by Dr. Ervant Arslau, in *Le Progrès Médical*: "In 1890, when I was assistant at the Children's Hospital in Padua, I had some experience in the use of the electric current in children's diarrhœa. Having observed that the Faradic current applied directly on the abdomen succeeded in checking the diarrhœa after a few applications, I treated many patients and obtained always good results. The intensity should be strong enough to produce visible contraction of the muscles. The current is very well endured and produces no pain, for several children remained motionless without uttering the slightest cry during the whole process. Generally after three or four applications the diarrhœa is suppressed, and at the same time other symptoms (fever, vomiting, want of appetite, &c.) which so often accompany this complaint are relieved. In July, 1892, I had occasion to treat two cases of cholera received at the hospital. These two cholera patients, one aged nine, the other twelve years, came from Suresnes, a region known to be infested with true Asiatic cholera. In both these patients the diarrhœa ceased after the first application of electricity. One of the patients, while cured of his diarrhœa, suffered still from bilious vomiting. The 'faradisation' of the pneumogastric nerve for a minute caused the vomiting to cease instantly. The gravity of the illness did not allow of confining the treatment in these cases solely to electricity, but the results obtained in all the others—about thirty in number—and the rapidity of its action on the two cholera patients, authorise us in asserting that our method, either alone or combined with other remedies, can render very great service in the treating of this terrible malady."

### SANITATION IN RUSSIA.

A writer in the *Edinburgh Review* attempts to show that the misery which lately broke upon the third part of European Russia was the result of a progressive impoverishment during the last twenty years. He has collected some striking facts bearing upon the recent epidemic and famine. He draws attention to the fact that the constitutions of the peasants are almost universally undermined by habitual drunkenness; 2,000 persons die annually in European Russia from delirium tremens. The temperance movement, though gaining strength in some parts, is not favoured by the Government, since twenty-five million sterling annually is derived from the Kabak or brandy tavern, found in every village. "To the Russian lower classes the idea of cleanliness is a chimera; they lie in abject filth and feel comfortable in it. A characteristic proverb says, 'The wolf and the bear never wash, yet are healthy.' Even apart from the cost of sanitation, the peasants would consider it a mere waste of time to clean their huts, in consequence, as Lanin says, of their failing entirely to grasp the relation between filth and contagion, so diseases never cease, and not only cholera, but typhus fever and diphtheria are constantly recurring, because the houses have cesspools under their floors and the rivers are polluted with offal. Like the famine, contagious disease is regarded by the moujik as God's infliction, to which he must submit, and against which only prayers and charms can work. He not only scorns medical aid, but rebels against it. Mob excesses are possible everywhere, but such excesses as were witnessed last summer at Astrachan and Saratov, where the populace burned sick men with petroleum on a pile, stormed the hospital and threw the physician into the river, are only possible in Russia. In one region the cholera was even ascribed to the machinations of English emissaries, who last year, under pretence of distributing alms to the hungry, visited the famine districts, and bribed the Russian physicians to spread the infection."

### WRITERS' CRAMP.

A case is recorded of a young man who for five years had suffered from writers' cramp, which had resisted all attempts at cure. Examining his hand, Benedikt discovered at the root of one of the joints a swelling, painful to the touch. At this swelling he administered injections with a solution of phenic acid, and thus obtained the disappearance of the cramp. Later he administered successfully phenic injections to a pianiste long troubled with professional cramp in one of her hands. In this case he had found, as in the preceding patient, at the bend of the fore-arm a painful swelling of one of the tendons of the hand. (*Wien. Med. Woch.*)

### IS OPIUM HURTFUL TO THE CHINESE?

The question is answered in the negative in two separate articles in the *Asiatic Quarterly Review*. In the one case we have the opinion of a Chinaman, who may be considered prejudiced. But Mr. A. P. Nightingale, M.B., C.M., arrives at the same conclusions as to the relative harmlessness of the drug as used by the Chinese. He says: "My experience is local and still limited, but after nearly a year's work among the Chinese in Johore and the examination of, and making notes on, the subject of over a thousand patients as they passed through the hospital, I am of opinion that not only is the use of opium not an evil, but that in the majority of cases it is even beneficial in warding off or lessening attacks of fever and in enabling a Chinaman to perform heavy coolie labour with the thermometer at 150 degrees, which no other race can do. If a man over-smokes himself—and one is astonished to find how few do so—he merely goes to sleep, and wakes again quite fresh. It never makes him quarrelsome; it enables him to stand a vast amount of pain, and is one of the very few pleasures of his life. Compare this with the use and abuse of alcohol. I really believe that the more the opium question is inquired into the less harm will be seen to result from the use of this drug."

